# An NIR-responsive FeS-loaded alginate hydrogel promotes diabetic wound healing through Nrf2 activation and macrophage polarization

**DOI:** 10.1016/j.ijpx.2026.100645

**Published:** 2026-08-21

**Authors:** Xiaofang Wang, Jun Chen, Tingyi Guo, Huisheng Yao, Nan Li, Zhenggang Li, Jia Li

**Affiliations:** a Department of Anesthesiology, Shengjing Hospital of China Medical University, Shenyang, China; b Department of Ophthalmology, The First Affiliated Hospital of China Medical University, Shenyang, Liaoning, China; c Department of Pediatrics, Shengjing Hospital of China Medical University, Shenyang, Liaoning, China; d Department of Pediatrics, The Fourth Affiliated Hospital of China Medical University, Shenyang City, Liaoning Province, 110032, China; e Department of Hematology, Shengjing Hospital of China Medical University, Shenyang, China

**Keywords:** Diabetic wound, Iron sulfide, Alginate hydrogel, Oxidative stress, Nrf2, Macrophage polarization

## Abstract

Diabetic wounds are characterized by excessive oxidative stress, persistent inflammation, and impaired tissue regeneration, which collectively hinder normal wound healing. Here, we developed a near-infrared (NIR)-responsive sodium alginate hydrogel incorporating nano‑iron sulfide (nFeS) through mild gelation mediated by calcium carbonate and glucono-δ-lactone. The resulting SA-nFeS hydrogel exhibited a porous structure, favorable rheological properties, hydrogen peroxide-responsive degradation, radical-scavenging activity, and good cytocompatibility. NIR irradiation accelerated the release of Fe from the hydrogel, increasing the cumulative release from approximately 35.5% to 75.4% over 48 h. In cells exposed to oxidative stress, the NIR-treated SA-nFeS hydrogel markedly reduced intracellular reactive oxygen species and promoted Nrf2 nuclear translocation, accompanied by decreased Keap1 expression and increased expression of the downstream antioxidant proteins HO-1, SOD1, SOD2, and GPX4. Furthermore, the hydrogel suppressed pro-inflammatory macrophage markers and cytokines while increasing the expression of CD206, Arg-1, and IL-10, indicating a shift toward a pro-healing macrophage phenotype. In a streptozotocin-induced diabetic full-thickness wound model, SA-nFeS combined with NIR irradiation achieved approximately 93% wound-area reduction by Day 14 and promoted re-epithelialization, granulation tissue formation, and collagen deposition. No obvious histopathological abnormalities were observed in the major organs. Collectively, these findings demonstrate that the NIR-responsive SA-nFeS hydrogel promotes diabetic wound repair by activating endogenous antioxidant defense and modulating the inflammatory microenvironment, providing a promising therapeutic platform for chronic wound management.

## Graphical abstract

**Figure:**
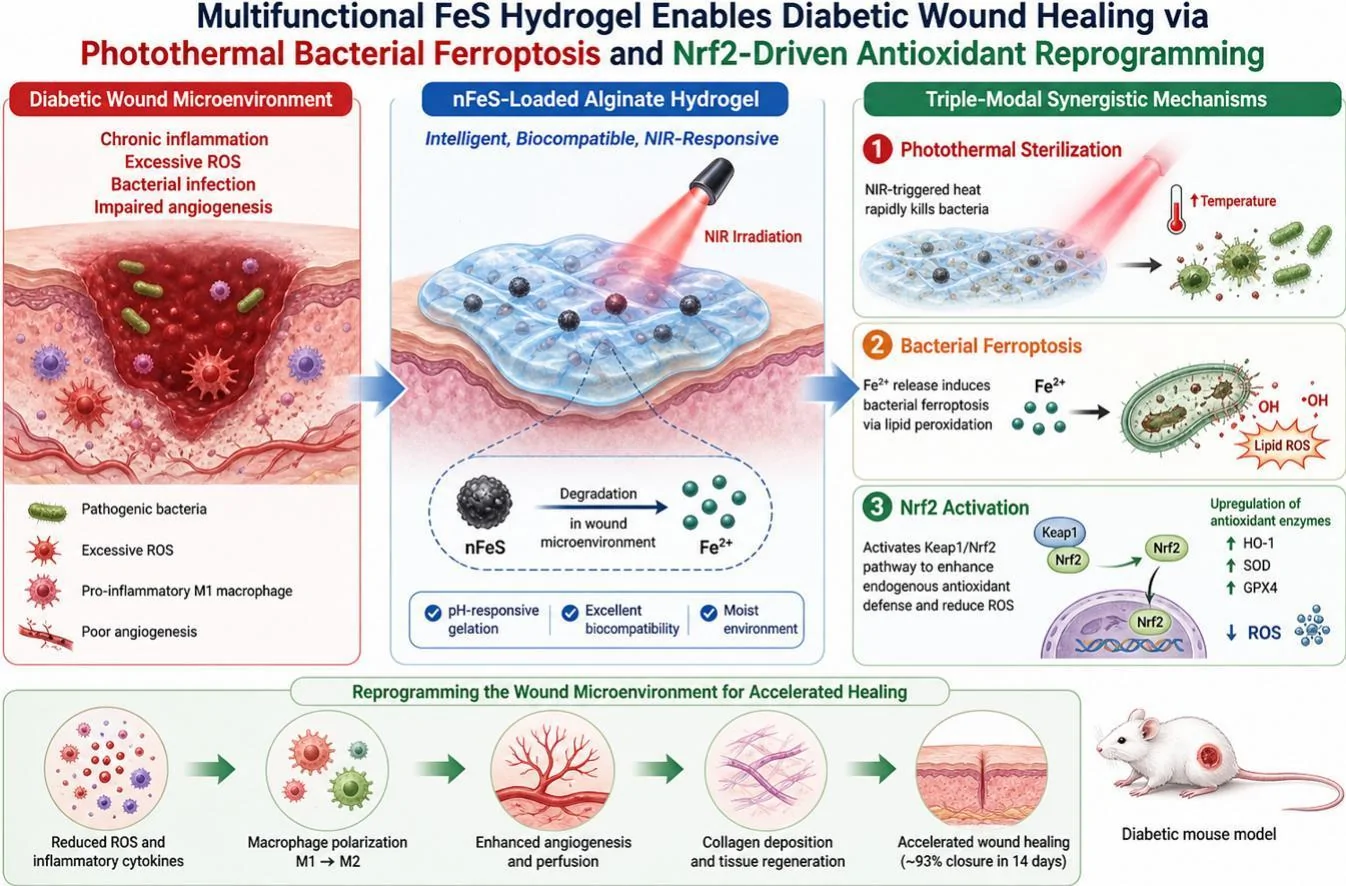

## 1 Introduction

Diabetes mellitus is a metabolic disorder characterized by persistent hyperglycemia, with its prevalence continuing to rise. It is estimated that by 2045, the number of diabetic patients worldwide will reach 700 million, posing a major public health challenge that threatens human health (Shi et al., 2024; Fang, 2018; Simon et al., 2021). Diabetic wounds, a frequent and complex complication of this disease, arise from the systemic disruption of the normal wound-healing process under hyperglycemic conditions, leading to the transition of acute wounds into chronic and non-healing ulcers. Prolonged inflammation is the primary obstacle to effective wound repair and remains a major clinical challenge (Cui et al., 2025; Liu et al., 2022; Jin et al., 2025). The pathological microenvironment of diabetic wounds is particularly complex and detrimental: sustained hyperglycemia not only damages vascular endothelial cells, but also promotes the accumulation of advanced glycation end products (AGEs) in local tissues, resulting in vascular stiffening, impaired microcirculation, and hindered nutrient and oxygen delivery to the wound site (González et al., 2023; Sun et al., 2025). Elevated levels of reactive oxygen species (ROS) disrupt redox homeostasis and induce persistent oxidative stress, which directly damages tissue cells and inhibits macrophage polarization toward the anti-inflammatory M2 phenotype, thereby trapping the wound in the inflammatory phase (Sun et al., 2025; Zhang et al., 2021; Zhao et al., 2020; Wei et al., 2024). This oxidative and inflammatory microenvironment ultimately impairs cellular proliferation, extracellular matrix remodeling, and tissue regeneration (Berthiaume et al., 2021; Li et al., 2018; Yang et al., 2024; Bei et al., 2024; Zhang et al., 2026). Therefore, the simultaneous regulation of excessive oxidative stress and persistent inflammation represents an important therapeutic strategy for diabetic wound repair.

Current clinical approaches for diabetic wounds include glycemic control, surgical debridement, revascularization when indicated, skin grafting, and dressing interventions (Borys et al., 2019; Wang and Zhang, 2024; Xin et al., 2023; Eriksson et al., 2022). Among these, dressings directly interface with the wound and critically influence healing outcomes, making the development of multifunctional dressings a key research focus. Hydrogels, with their three-dimensional porous architecture, high water content, and excellent biocompatibility, offer unique advantages in absorbing exudate, maintaining a moist environment, protecting the wound surface, and serving as drug delivery platforms (Li et al., 2025a; Li et al., 2025b; Tong et al., 2022; Alven et al., 2022; Li et al., 2026). While many studies have incorporated antioxidant compounds, including natural polyphenols and flavonoids, into hydrogel systems to alleviate oxidative stress (Zeng et al., 2025), many exogenous antioxidants provide only transient radical-scavenging effects and may be rapidly consumed in the highly oxidative diabetic wound microenvironment. Notably, ROS act as a “double-edged sword” in wound repair: physiological levels support cellular signaling, immune responses, and tissue repair, whereas excessive ROS drive persistent inflammation and tissue damage (Qu et al., 2024). Thus, an effective diabetic wound dressing should not simply eliminate ROS indiscriminately, but should restore redox homeostasis while reinforcing endogenous cellular antioxidant defenses (Luo et al., 2026).

The Kelch-like ECH-associated protein 1/nuclear factor erythroid 2-related factor 2 (Keap1/Nrf2) signaling pathway is a central regulator of oxidative stress responses (Li et al., 2026, Liao et al., 2025, Liao et al., 2025, Wang et al., 2025). Under normal conditions, Nrf2 is bound to Keap1 and is continuously targeted for degradation in the cytoplasm. In response to oxidative or electrophilic stimuli, the interaction between Keap1 and Nrf2 is disrupted, allowing stabilized Nrf2 to accumulate and translocate into the nucleus (Shi et al., 2025, Xie et al., 2025, Zang et al., 2025, Zhang et al., 2025, Feng et al., 2026). Nuclear Nrf2 subsequently promotes the transcription of cytoprotective and antioxidant genes, including those encoding heme oxygenase-1 (HO-1), superoxide dismutases (SODs), glutathione-related enzymes, and other phase II detoxification proteins (Mesfin et al., 2025). Activation of this endogenous antioxidant network may provide a more sustained response to oxidative stress than the direct administration of a single exogenous antioxidant (Liao et al., 2023, Liao et al., 2024, Liao et al., 2026, Liao et al., 2026, Liu et al., 2025). Furthermore, attenuation of oxidative stress may suppress persistent inflammatory activation and facilitate macrophage transition toward a pro-healing phenotype, thereby creating a microenvironment favorable for tissue regeneration.

Nano‑iron sulfide (nFeS) has emerged as a promising functional nanomaterial because of its physicochemical responsiveness and potential biological activity. Previous studies have reported that nanoscale iron sulfide can release reduced sulfur species or polysulfide-related components under physiological conditions. These species may modify redox-sensitive cysteine residues on Keap1, thereby facilitating Nrf2 stabilization and nuclear translocation (Wang et al., 2025). In addition, nFeS exhibits intrinsic radical-scavenging activity and can undergo accelerated degradation under oxidative conditions. Its photothermal response to near-infrared (NIR) irradiation may also provide an externally controllable means of modulating material degradation and the release of iron-containing components (Liao et al., 2026, Liao et al., 2026, Liu et al., 2026, Xu et al., 2025, Yang et al., 2024). These properties suggest that nFeS may combine direct ROS-scavenging activity with activation of endogenous antioxidant defense. Nevertheless, the direct application of free nanoparticles may be limited by insufficient local retention, dispersion instability, and uncontrolled exposure, highlighting the need for an appropriate biomaterial carrier (Liu et al., 2025).

In the present study, we developed an NIR-responsive nFeS-loaded sodium alginate hydrogel for diabetic wound treatment. The hydrogel is based on sodium alginate, offering excellent biocompatibility and biodegradability. Calcium carbonate (CaCO₃) and glucono-δ-lactone (GDL) serve as crosslinkers; the gradual release of H^+^ from GDL triggers Ca^2+^ release, enabling mild internal gelation and avoiding the potential irritation associated with conventional chemical crosslinkers. The incorporation of nFeS was intended to provide hydrogen peroxide-responsive degradation, radical-scavenging activity, and NIR-accelerated release of iron-containing components. We systematically evaluated the physicochemical properties, cytocompatibility, antioxidant activity, and cellular effects of the resulting SA-nFeS hydrogel. Particular attention was paid to intracellular ROS regulation, activation of the Keap1/Nrf2 antioxidant pathway, and modulation of macrophage-associated inflammatory phenotypes. Finally, its therapeutic efficacy and histological safety were assessed in a streptozotocin-induced diabetic full-thickness wound model. We hypothesized that the SA-nFeS hydrogel would promote diabetic wound repair by integrating direct ROS attenuation, endogenous antioxidant activation, and inflammatory microenvironment modulation.

## 2 Materials and Methods

### 2.1 Materials

All chemicals were of analytical grade and used without further purification. Iron(III) chloride hexahydrate (FeCl₃·6H₂O), L-cysteine, sodium alginate, calcium carbonate (CaCO₃), glucono-δ-lactone (GDL), 2,2-diphenyl-1-picrylhydrazyl (DPPH), and 2,2′-azinobis(3-ethylbenzothiazoline-6-sulfonic acid) diammonium salt (ABTS) were purchased from Sigma-Aldrich (USA). Potassium persulfate (K₂S₂O₈) was obtained from Aladdin (China). Phosphate-buffered saline (PBS, 1×, pH 7.4) and Dulbecco's modified Eagle's medium (DMEM) were supplied by Gibco (USA). Hydrogen peroxide (H₂O₂, 30 wt%) was purchased from Sinopharm Chemical Reagent Co., Ltd. (China). Deionized water (DI water) with a resistivity of 18.2 MΩ·cm was produced using a Milli-Q water purification system.

### 2.2 Preparation and characterization of nFeS nanoparticles

nFeS nanoparticles were prepared *via* a hydrothermal method (Zeng et al., 2025). Briefly, Fe^3+^ was used as the iron source, and cysteine served as both the sulfur source and coordinating ligand. The reaction was carried out at 120 °C for 6 h under high-pressure conditions. The morphology of nFeS nanoparticles was observed using field emission scanning electron microscopy (FE-SEM, Hitachi Regulus 8100) at an accelerating voltage of 15 kV. The hydrodynamic diameter of nFeS nanoparticles was determined by dynamic light scattering (DLS) using a Malvern Zetasizer Nano ZS instrument. Measurements were performed on aqueous dispersions at a concentration of 200 μg/mL and a temperature of 25 °C. The reported value represents the *Z*-average diameter derived from the intensity-weighted distribution. The long-term colloidal stability of nFeS nanoparticles was investigated in DI water, PBS, and cell culture medium over 7 days. Samples (200 μg/mL) were incubated at 37 °C, and their hydrodynamic size (Z-average) and PDI were measured by DLS at selected time points.

### 2.3 Preparation of hydrogels

To prepare the composite hydrogels, 5, 10, or 15 mg of nFeS nanoparticles were dispersed in 10 mL of sodium alginate solution (1.25 wt%), corresponding to final nFeS concentrations of 0.5, 1.0, and 1.5 mg/mL, respectively. These concentrations were selected as an exploratory formulation range to evaluate the loading-dependent effects of nFeS on the physicochemical and functional properties of the hydrogel. Subsequently, 11 mg of calcium carbonate and 0.1 g of glucono-δ-lactone were added to initiate internal gelation, and the mixtures were stirred to ensure homogeneous gel formation. Based on the overall balance among mechanical stability, oxidative responsiveness, radical-scavenging activity, NIR responsiveness, and cytocompatibility, the hydrogel containing 1.0 mg/mL nFeS was selected for the subsequent cellular and *in vivo* experiments unless otherwise specified.

### 2.4 Photo-responsive release of iron ions

To evaluate the triggering effect of near-infrared (NIR) laser irradiation on the release behavior of Fe, nFeS-loaded hydrogel samples were immersed in PBS release medium. The experimental group was exposed to 808 nm NIR laser irradiation (power density: 1.0 W/cm^2^, 2 min), while the control group was kept under identical conditions without light exposure. Samples were collected periodically over 48 h, and the concentration of released Fe in the medium was determined by inductively coupled plasma optical emission spectrometry (ICP-OES) to calculate the cumulative release percentage (*n* = 3).

### 2.5 Photothermal performance and cycling stability test

To evaluate the photothermal conversion performance of nFeS nanoparticles, dispersions at different concentrations (0, 0.5, 1.0 and 1.5 mg/mL) were placed in quartz cuvettes and irradiated vertically with an 808 nm near-infrared laser (power density: 1.0 W·cm^−2^) for 2 min. Temperature changes were recorded and thermal images were captured simultaneously using an infrared thermal camera. Three parallel samples were prepared for each concentration, and ΔT was calculated as the difference between the final temperature at the end of irradiation and the initial temperature (25 °C).

For the photothermal cycling stability test, a 1.0 mg/mL nFeS dispersion was subjected to five consecutive cycles of 2 min irradiation followed by natural cooling to room temperature under the same laser parameters. Temperature variations during each cycle were continuously monitored by a thermocouple, and performance attenuation was assessed *via* temperature-time curves. All data are presented as the mean ± standard deviation of three independent experiments.

### 2.6 Rheological characterization of hydrogels

Rheological properties of the hydrogels were evaluated using a rotational rheometer (Discovery HR 20). Storage modulus (G′) and loss modulus (G″) were measured as functions of frequency and time under controlled strain conditions. Two distinct testing modes were performed: Dynamic frequency sweep: at a constant strain of 0.5%, with a frequency range from 0.01 to 10 Hz. Strain amplitude sweep: at a constant frequency of 1 Hz, with a strain range from 0.01% to 100%. All tests were conducted using a fixture with a diameter of 40 mm.

### 2.7 SEM imaging of hydrogels

The microstructure of the lyophilized hydrogels was examined by FE-SEM (Hitachi Regulus 8100). Samples were freeze-dried and sputter-coated with gold prior to imaging.

### 2.8 Swelling and stability studies

Swelling behavior was assessed by immersing 0.5 g of hydrogel in twice its volume of aqueous medium. The swelling ratio was calculated at predetermined time intervals until equilibrium was reached. Stability tests were conducted in phosphate-buffered saline (PBS, pH 7.4) and H₂O₂ (0.5 mM, 1 mM, 10 mM) solution to simulate normal and oxidative pathological environments, respectively.

### 2.9 *In vitro* ROS scavenging assay

The antioxidant capacity of the hydrogels was evaluated using the DPPH assay and ABTS assay. Briefly, 50 mg of hydrogel was incubated in 0.5 mL of DPPH solution (0.2 mg/mL). The scavenging efficiency was measured at various time points by monitoring the absorbance decrease at 517 nm. After 24 h of reaction, the absorbance at 517 nm was measured and denoted as A_x_. A control group using PBS instead of the hydrogel was measured under the same conditions and denoted as A_0._ Scavenging efficiency (%) was calculated as follows:

$$\text{Scavenging efficiency}\;\left(\%\right)=\left(1-A_{x}/A_{0}\right)\times 100\%$$

The ABTS assay was performed by preparing 7.4 mmol/L ABTS solution and 2.6 mmol/L potassium persulfate solution, which were mixed at a 1:1 (*v*/v) ratio and allowed to react in the dark for 12 h. The resulting mixture was then diluted 50-fold to obtain the ABTS working solution. Subsequently, 1 mL of the ABTS working solution was added to 0.5 g of hydrogel. After 24 h of reaction, the absorbance at 734 nm was measured and denoted as A_x_. A control group using PBS instead of the hydrogel was measured under the same conditions and denoted as A_0_. The ABTS scavenging efficiency (%) was calculated as follows:

$$\text{Scavenging efficiency}\;\left(\%\right)=\left(1-A_{x}/A_{0}\right)\times 100\%$$

### 2.10 Cell culture and hydrogel extract preparation

Murine fibroblasts (L929) and murine macrophages (RAW264.7) were cultured in Dulbecco's Modified Eagle Medium supplemented with 10% fetal bovine serum and 1% penicillin–streptomycin at 37 °C in a humidified atmosphere containing 5% CO₂. The SA-nFeS hydrogel used in the subsequent cellular experiments contained 1.0 mg/mL nFeS. To prepare hydrogel extracts, SA or SA-nFeS hydrogels were immersed in complete culture medium at a ratio of 0.1 g/mL and incubated at 37 °C for 24 h. The supernatants were collected and filtered through a 0.22 μm membrane before use. For the SA-nFeS + L group, the hydrogel was irradiated with an 808 nm NIR laser at 1.0 W/cm^2^ for 2 min before preparation of the extract. Unless otherwise specified, the resulting hydrogel extracts were used for all subsequent *in vitro* cellular experiments.

### 2.11 Live/dead cell viability assay

The cytocompatibility of the hydrogels was evaluated using a LIVE/DEAD™ Viability/Cytotoxicity Kit (Invitrogen, Thermo Fisher Scientific, USA). L929 cells were seeded into 96-well plates at a density of 1*10^4^ cells/well. After 24 h of attachment, the medium was replaced with the different hydrogel extracts. Following another 24 h of incubation, the cells were washed with PBS and stained with the Live/Dead working solution (2 μM calcein AM and 4 μM ethidium homodimer-1) for 20 min at 37 °C in the dark. The live (green) and dead (red) cells were observed and imaged using an inverted fluorescence microscope (Zeiss, Germany).

### 2.12 BrdU cell proliferation assay

Cellular proliferation was assessed by detecting DNA synthesis using a BrdU Cell Proliferation Assay Kit (Abcam, UK). L929 cells were cultured with the various hydrogel extracts for 24 h. Subsequently, BrdU labeling solution was added to the culture medium and incubated for 4 h. After fixation and permeabilization, the cells were incubated with an anti-BrdU primary antibody, followed by a fluorophore-conjugated secondary antibody. Cell nuclei were counterstained with DAPI (Sigma-Aldrich, USA). The ratio of BrdU-positive cells was quantified using ImageJ software (NIH, USA).

### 2.13 *In vitro* scratch wound healing assay

L929 cells were seeded in 6-well plates and cultured until they formed a confluent monolayer. A sterile 200 μL pipette tip was used to create a straight artificial scratch wound in each well. The detached cells were gently washed away with PBS, and the cells were then incubated with different hydrogel extracts. The migration of cells into the scratched area was monitored, and representative optical images were captured at 0 h and 24 h post-scratching using an inverted microscope.

### 2.14 Intracellular ROS scavenging assay

To evaluate the ROS-scavenging capability of the hydrogels, L929 cells were pre-treated with 200 μM H_2_O_2_ for 4 h to simulate a high-oxidative-stress environment. The cells were then treated with the respective hydrogel extracts for 12 h. Intracellular ROS levels were detected using a DCFH-DA fluorescent probe (Sigma-Aldrich, USA). The cells were incubated with 10 μM DCFH-DA for 20 min at 37 °C, washed with PBS, and immediately observed under a fluorescence microscope.

### 2.15 *In vitro* immunofluorescence staining

To evaluate Nrf2 nuclear translocation and macrophage polarization (CD86/CD206), cells subjected to corresponding treatments were fixed with 4% paraformaldehyde for 15 min, permeabilized with 0.1% Triton X-100 for 10 min, and blocked with 5% BSA for 1 h at room temperature. The cells were then incubated overnight at 4 °C with specific primary antibodies: anti-Nrf2 (Abcam, 1:200), anti-CD86 (Abcam, 1:200), or anti-CD206 (Abcam, 1:200). The next day, cells were incubated with Alexa Fluor 488/594-conjugated secondary antibodies (Invitrogen, USA, 1:500) for 1 h in the dark. Nuclei and cytoskeletal F-actin were stained with DAPI and Phalloidin-iFluor 594 (Abcam, UK), respectively.

### 2.16 Western blot analysis

Total protein from the treated cells was extracted using RIPA lysis buffer containing protease and phosphatase inhibitor cocktails (Thermo Fisher Scientific, USA). Protein concentrations were determined using a Pierce™ BCA Protein Assay Kit (Thermo Fisher Scientific, USA). Equal amounts of protein (30 μg) were separated by SDS-PAGE and transferred onto PVDF membranes (Millipore, USA). After blocking with 5% non-fat milk, the membranes were probed with primary antibodies against Nrf2, Keap1, HO-1, SOD1, SOD2, GPX4, and GAPDH (all from Cell Signaling Technology or Abcam, 1:1000 dilution) overnight at 4 °C. Following incubation with HRP-conjugated secondary antibodies (1:5000), the protein bands were visualized using an ECL detection kit (Thermo Fisher Scientific, USA) and analyzed with ImageJ software.

### 2.17 Enzyme-Linked Immunosorbent Assay (ELISA)

RAW264.7 macrophages were stimulated with 1 μg/mL LPS (Sigma-Aldrich, USA) to mimic an inflammatory state and treated with various hydrogel extracts. After 24 h, the cell culture supernatants were collected and centrifuged to remove cellular debris. The secretion levels of pro-inflammatory (IL-6) and anti-inflammatory (IL-10) cytokines were quantified using specific Mouse ELISA kits (R&D Systems, USA) according to the manufacturer's instructions.

### 2.18 Quantitative Real-Time PCR (RT-qPCR)

Total RNA was extracted from the treated macrophages or wound tissues using TRIzol reagent (Invitrogen, USA). The RNA was reverse-transcribed into cDNA using the PrimeScript™ RT reagent Kit (Takara Bio, Japan). RT-qPCR was performed using SYBR® Premix Ex Taq™ (Takara Bio, Japan) on an ABI 7500 Real-Time PCR System (Applied Biosystems, USA). The relative mRNA expression levels of target genes were calculated using the 2^-∆∆Ct^ method, with GAPDH serving as the endogenous control. The Primer sequences are shown in Table S1.

### 2.19 *In vivo* diabetic wound healing model

Diabetes was induced in 6–8-week-old male C57BL/6 mice by intraperitoneal injection of streptozotocin (STZ, Sigma-Aldrich, USA) at a dose of 50 mg/kg for 5 consecutive days. Mice with stable fasting blood glucose >16.7 mmol/L were selected. A full-thickness circular wound (diameter = 10 mm) was surgically created on the dorsum. No exogenous bacteria were inoculated into the wounds. Mice were randomly assigned to four groups (*n* = 5): Control, SA, SA-nFeS, and SA-nFeS + L. For the photothermal group, the wounds were irradiated with an 808 nm laser (1.0 W/cm^2^, 2 min). Digital photographs of the wounds were taken on Days 0, 3, 7, 10, and 14 to calculate the wound closure rate.

### 2.20 Histological and *in vivo* immunofluorescence analyses

Wound tissues were harvested on Day 7 and Day 14, fixed in 4% paraformaldehyde, embedded in paraffin, and sectioned (5 μm). The sections were stained with Hematoxylin and Eosin (H&E) and Masson's trichrome to evaluate epidermal regeneration, granulation, and collagen deposition. For *in vivo* mechanisms, sections were deparaffinized, subjected to antigen retrieval, and stained with primary antibodies against CD86, CD206, Nrf2, and HO-1, followed by fluorescent secondary antibodies and DAPI.

### 2.21 Systemic biosafety evaluation

To assess the *in vivo* systemic toxicity of the hydrogels, major organs including the heart, liver, spleen, lung, and kidney were collected from the mice at the end of the 14-day treatment period. The organs were sectioned and stained with H&E and Masson's trichrome to detect any morphological abnormalities, inflammatory infiltration, or fibrotic lesions.

### 2.22 Statistical analysis

All data are expressed as the mean ± standard deviation (SD). Statistical analyses were performed using GraphPad Prism 9.0 software (GraphPad Software, USA). One-way analysis of variance (ANOVA) followed by Tukey's multiple comparison test was used to determine the statistical significance among different groups. A *p*-value <0.05 was considered statistically significant (* *p* < 0.05, ** *p* < 0.01, *** *p* < 0.001, **** *p* < 0.0001).

## 3 Results and discussion

### 3.1 Synthesis and characterization

**Fig. 1:**
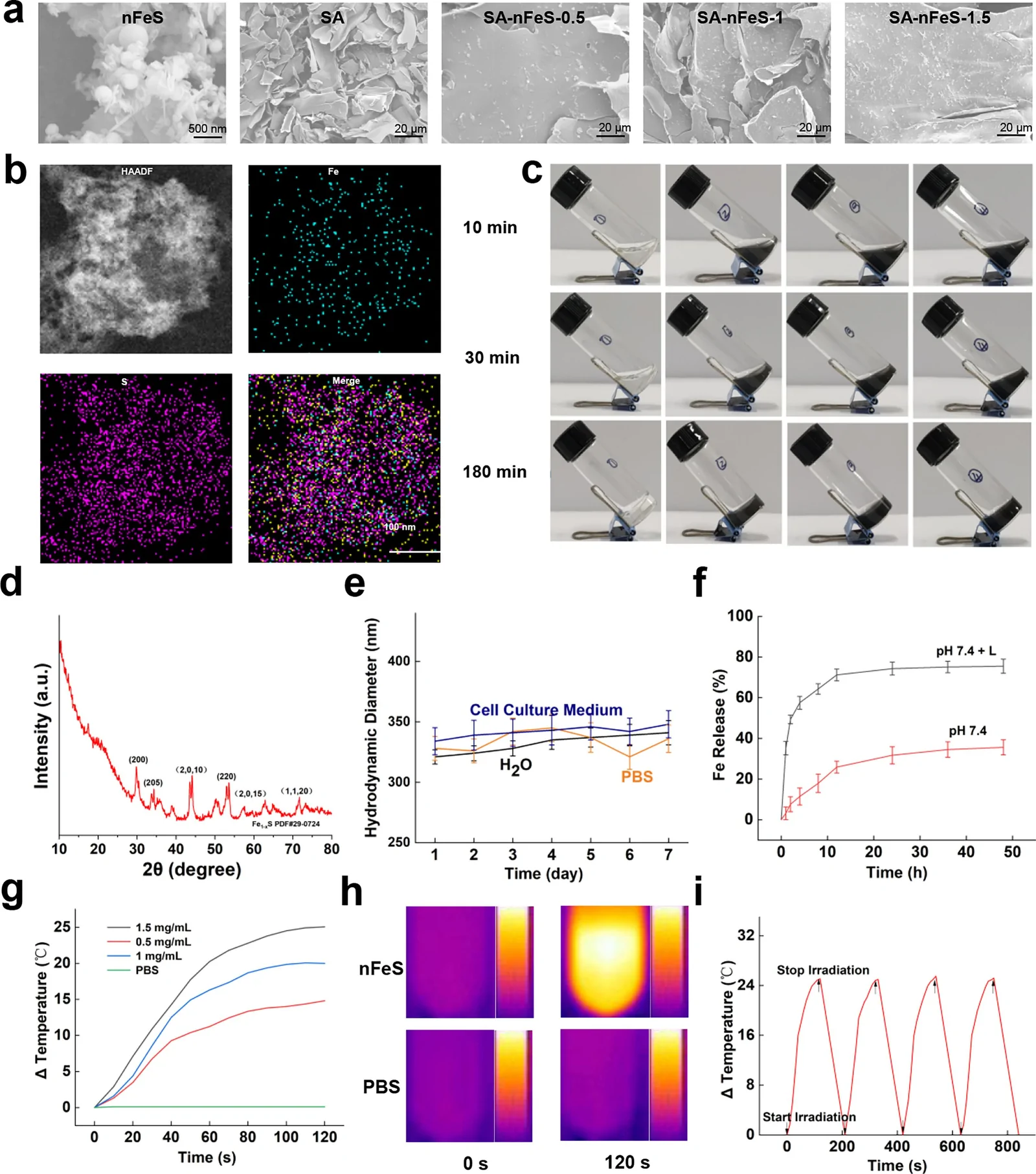
Physicochemical Characterization of FeS-GP gel. (a) Field emission scanning electron microscopy (FE-SEM) images depicting the morphology of synthesized nFeS nanoparticles, hydrogels and FeS-GP gel. (b) EDS elemental mapping analysis of FeS nanoparticles. (c) Photographs of hydrogels at different time points (10 min, 30 min, 180 min), illustrating the gelation process over time. (d) X-ray diffraction (XRD) pattern of nFeS nanoparticles, with the reference PDF#29–0724 for phase analysis; the indexed diffraction peaks correspond to specific crystal planes of nFeS. (e) Hydrodynamic size of the nFeS nanoparticles dispersed in water, PBS and cell culture medium as a function of storage time period. (f) Cumulative release profile of Fe from the FeS-GP gel with or without an 808 nm laser irradiation (1 W cm^−2^, 2 min) for each time point (*n* = 3) (g). Temperature change (Δ*T*) of the aqueous suspension of the nFeS particles under laser irradiation for 2 min. (h) Temperature elevation plot of PBS and the aqueous suspension of nFeS particles under an 808-nm laser irradiation (1 W/cm^2^, 2 min). (i) Temperature plot of the aqueous suspension of nFeS particles (nFeS = 1.0 mg/mL) during five cycles of laser on-off (laser on: 120 s; laser off: cool to room temperature).

This study successfully constructed an intelligent sodium alginate-based hydrogel integrated with nano iron sulfide (nFeS) for diabetic wound management. The hydrogel is formed *via* mild pH-responsive gelation mediated by calcium carbonate and glucono-δ-lactone, ensuring excellent biocompatibility and environmental adaptivity. SEM images revealed that the as-prepared nFeS exhibited a uniformly dispersed structure with relatively homogeneous dimensions (Fig. 1a). Furthermore, the FeS nanoparticles were observed to be uniformly distributed on the gel. TEM-EDS compositional analysis confirmed that the nanoparticles consist of uniformly distributed iron and sulfur, successfully forming pure FeS nanoparticles (Fig. 1b). Macroscopic morphology photographs of the hydrogels (Fig. 1c) at different time points (10, 30, and 180 min) visually document their gelation process, demonstrating a transition from a flowable sol to a stable gel state, which indicates a significant enhancement in crosslinking density and structural rigidity over time. X-ray Diffractometer (XRD) analysis was performed to further confirm the structure of nFeS. As shown in Fig. 1d, the XRD pattern of nFeS displayed a series of sharp characteristic diffraction peaks at 2θ = 30.0°, 34.4°, 44.2°and 53.6°, consistent with previously reported crystal structures, confirming its successful synthesis (Zeng et al., 2025). DLS analysis indicated that the hydrodynamic diameters of nFeS was approximately 290 nm with a narrow distribution (Fig. S1). The colloidal stability of the nFeS nanoparticles dispersed in different aqueous media (H_2_O, PBS, or cell culture medium with 10% fetal bovine serum) was investigated through measuring their hydrodynamic size for 7 days. Obviously, the hydrodynamic size of the particles does not show any significant changes within a given time period (Fig. 1e), thus validating their good colloidal stability. We further investigated the effect of near-infrared (NIR) laser irradiation on the release behavior of Fe. The results demonstrated that light exposure significantly promoted Fe release: within 48 h, the accumulative release rate in the laser-irradiated group reached over 75.4%, whereas that in the non-irradiated control group was only about 35.5% (Fig. 1f). These findings indicate that NIR laser irradiation can effectively trigger and accelerate the release of Fe through its photothermal effect. Subsequently, we evaluated the photothermal conversion performance of nFeS nanoparticles. As expected, under 808 nm laser irradiation, the temperature of nFeS nanoparticles increased with rising concentration (Fig. 1g). When the concentrations of nFeS nanoparticles were 0, 0.5, 1, and 1.5 mg·mL^−1^, the corresponding ΔT values were 0.1, 14.5, 19.6, and 24.4 °C, respectively corresponds well with the thermal imaging (Fig. 1h). To further verify the photothermal stability of nFeS nanoparticles, five consecutive cycles of near-infrared laser irradiation (laser on: power density 1 W·cm^−2^, duration 2 min) and natural cooling (laser off) were performed on its suspension at a concentration of 1 mg·mL^−1^ (Fig. 1i). Clearly, no significant performance attenuation was observed during the laser on-off cycles, indicating that the material possesses favorable photothermal stability and promising potential as a photothermal therapy agent.

### 3.2 Physicochemical characterization of FeS-GP gel

#### 3.2.1 Swelling and degradation behavior

**Fig. 2:**
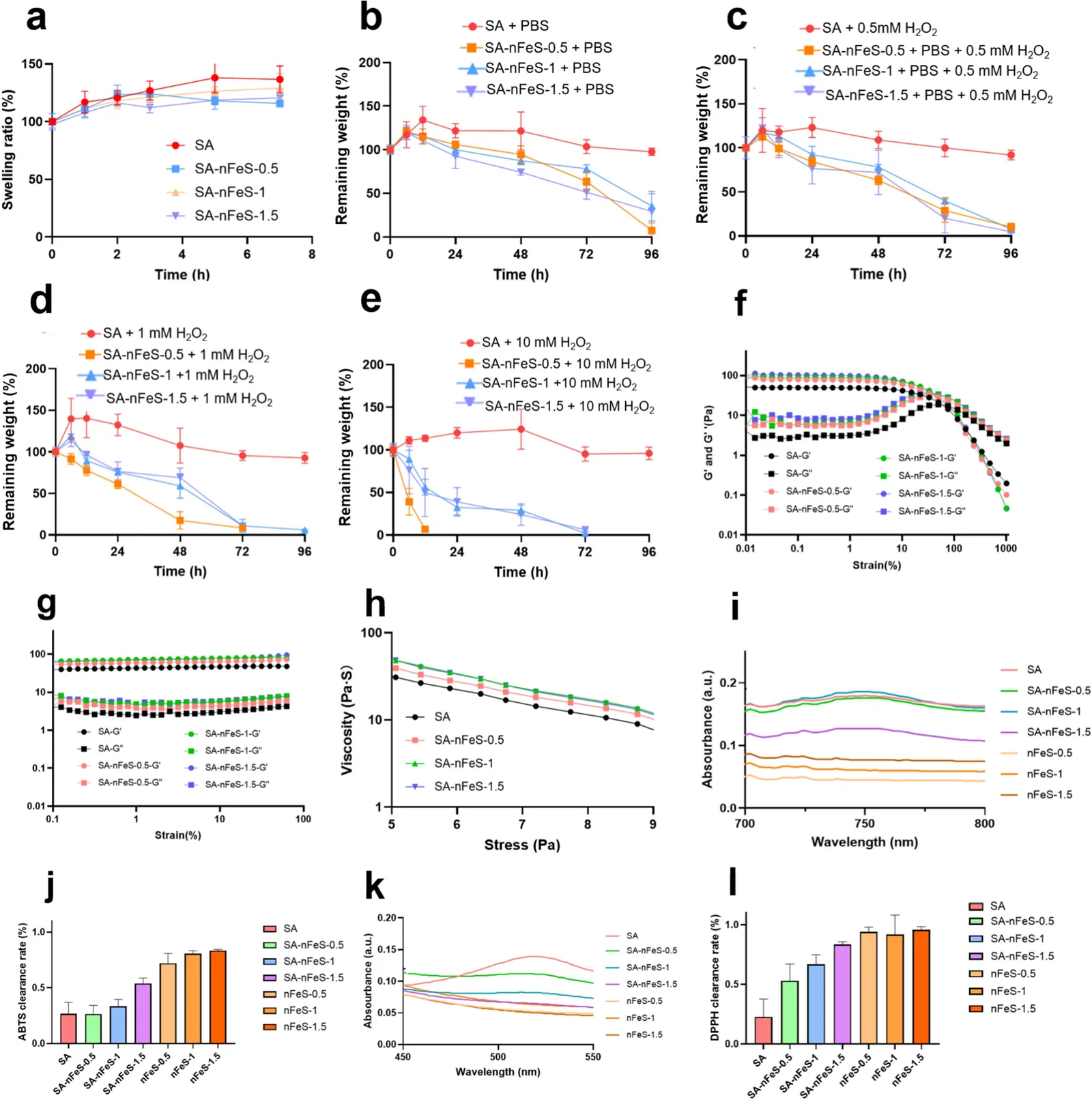
Physicochemical characterization of hydrogels. (a) Swelling behavior of the hydrogel in aqueous medium. (b) Stability assessment of the hydrogel in PBS at different nFeS concentrations over time. (c) Stability assessment of the hydrogel in 0.5 mM H₂O₂-containing PBS with varied nFeS contents over time. (d) Residual weight of SA and SA/nFeS hydrogels in 1 mM H₂O₂-containing PBS with different nFeS concentrations over time. (e) Residual weight of SA and SA-nFeS hydrogels in 10 mM H₂O₂-containing PBS with varied nFeS loadings over time. (f) Stress-strain curves of SA and SA-nFeS hydrogels with different nFeS contents. (g) Storage modulus (*G*′) and loss modulus (*G*″) of SA hydrogel, SA-nFeS hydrogels, and nFeS dispersions as a function of strain. (h) Viscosity of SA and SA-nFeS hydrogels with different nFeS loadings as a function of stress. (i) UV–Vis absorption spectra of SA hydrogel, SA-nFeS hydrogels, and nFeS dispersions with varied nFeS contents. (j) ABTS radical scavenging activity of SA hydrogel, SA-nFeS hydrogels, and nFeS dispersions with different nFeS concentrations. (k) UV–Vis absorption spectra of DPPH radical scavenging by SA hydrogel, SA-nFeS hydrogels, and nFeS dispersions with varied nFeS loadings. (l) The residual DPPH concentrations measured after the assay for SA hydrogel, SA-nFeS hydrogels, and nFeS dispersions at different nFeS loadings.

The swelling ratio of SA hydrogels was moderately affected by nFeS incorporation (Fig. 2a), implying that nFeS has a limited impact on the water-uptake capacity of SA matrices. SA-nFeS hydrogels exhibited enhanced stability in PBS with increasing nFeS content (Fig. 2b), suggesting that nFeS reinforces SA hydrogels against hydrolytic break down. H₂O₂-Responsive Degradation: In H₂O₂-containing media (Fig. 2c–e), degradation rates increased with both H₂O₂ concentration and nFeS loading. This H₂O₂-responsive behavior highlights the potential of SA-nFeS hydrogels for stimuli-responsive biomedical applications (*e.g.*, targeted drug delivery).

#### 3.2.2 Mechanical and rheological properties

Stress-strain curves showed that SA-nFeS hydrogels possess higher mechanical strength (stress at break) than pure SA hydrogel, with strength increasing with nFeS content (Fig. 2f). Rheological analysis revealed elevated storage modulus (G′) in SA-nFeS hydrogels (Fig. 2g), indicating improved viscoelasticity. The shear-thinning behavior supports their potential as injectable formulations (Fig. 2h, decreasing viscosity with increasing stress).

#### 3.2.3 Antioxidant

UV–Vis absorption spectra of SA hydrogel, SA-nFeS hydrogels, nFeS dispersions with varied nFeS contents, ABTS (Fig. 2i-j) and DPPH (Fig. 2k-l) assays demonstrated concentration-dependent antioxidant activity in SA-nFeS hydrogels, attributed to the ROS-scavenging capacity of nFeS (Fig. S2-S3). This makes them promising for antioxidant therapy or wound healing. The DPPH photodegradation assay demonstrated that the SA-nFeS hydrogels can scavenge ROS, with the scavenging efficiency increasing with higher nFeS loading.

### 3.3 *In vitro* biocompatibility, cell proliferation, and migration behavior

**Fig. 3:**
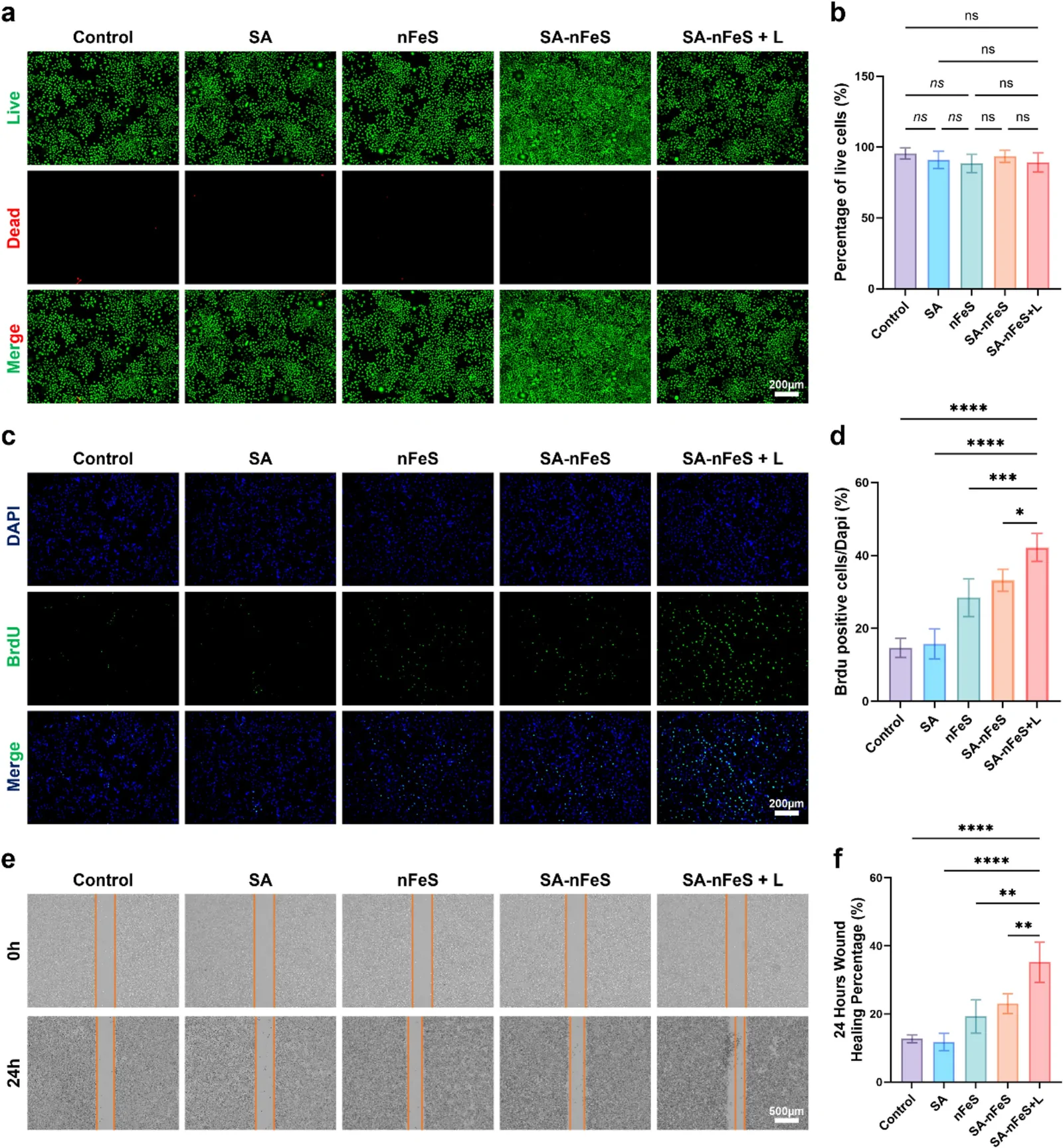
*In vitro* biocompatibility, cell proliferation, and migration evaluations of the hydrogels on L929 cells. (a) Representative fluorescence images of Live/Dead staining of L929 cells following incubation with different formulations (green represents live cells, red represents dead cells). Scale bar = 200 μm. (b) Quantitative analysis of the cell viability (percentage of live cells) based on the Live/Dead staining results. (c) Representative fluorescence images of the BrdU incorporation assay used to assess DNA synthesis and cellular proliferation (blue: DAPI-stained nuclei, green: BrdU-positive proliferating cells). Scale bar = 200 μm. (d) Quantification of the percentage of BrdU-positive cells relative to the total number of cells. (e) Representative optical microscope images of the *in vitro* scratch wound healing assay at 0 h and 24 h post-treatment, showing the migration capacity of L929 cells. Scale bar = 500 μm. (f) Quantitative analysis of the wound healing percentage after 24 h of incubation. (Data are presented as mean ± standard deviation. ns: no significant difference; *p < 0.05, **p < 0.01, ***p < 0.001, ****p < 0.0001). (For interpretation of the references to colour in this figure legend, the reader is referred to the web version of this article.)

Excellent biocompatibility is a fundamental prerequisite for the clinical application of any wound dressing. To evaluate the cytotoxicity of the hydrogels, a Live/Dead staining assay was performed on L929 cells following co-incubation with different formulations. As shown in Fig. 3a, widespread green fluorescence (live cells) and negligible red fluorescence (dead cells) were observed across all experimental groups. Quantitative analysis confirmed that cell viability remained above 90% in all groups, exhibiting no significant statistical difference (ns) compared to the control group (Fig. 3b). Notably, treatment with the extract derived from the NIR-irradiated SA-nFeS hydrogel did not significantly reduce L929 cell viability compared with the control group. This finding supports the cytocompatibility of the SA-nFeS formulation after NIR treatment under the tested experimental conditions. However, because the cells were not directly exposed during laser irradiation, these results do not provide direct evidence regarding thermal injury or the *in situ* thermal safety of the treatment.

Beyond basic biocompatibility, promoting cellular proliferation and migration is crucial for accelerating the re-epithelialization of chronic diabetic wounds. A BrdU incorporation assay was utilized to assess DNA synthesis and cellular proliferation (Fig. 3c). The results revealed a marked increase in BrdU-positive cells (green fluorescence) in the nFeS-containing groups compared to the pure SA and control groups. Quantitative datafurther demonstrated that the SA-nFeS + L group achieved the highest cell proliferation rate (approximately 42%), which was significantly higher than that of the non-irradiated SA-nFeS group (*** *p* < 0.001) and the control group (**** *p* < 0.0001) (Fig. 3d). These findings indicate that treatment with the SA-nFeS formulation, particularly after NIR irradiation, was associated with enhanced L929 cell proliferation under the tested conditions.

Furthermore, an *in vitro* scratch wound healing assay was conducted to evaluate the effect of the hydrogels on cell migration dynamics (Fig. 3e). After 24 h of incubation, the SA-nFeS + L group exhibited the most rapid scratch closure among all treatment groups. As quantified in Fig. 3f, the wound healing rate of the SA-nFeS + L group reached approximately 35%, which was substantially greater than that of the control group (∼12%) and the SA-nFeS group (∼22%) (** *p* < 0.01). This significantly enhanced migratory capacity can be attributed to the continuous modulation of the microenvironment by the nFeS hydrogel under light irradiation, a remodeling effect that likely triggers downstream signaling pathways favorable for L929 cell motility and tissue regeneration.

### 3.4 Intracellular ROS scavenging and activation of the Keap1/Nrf2 antioxidant pathway

**Fig. 4:**
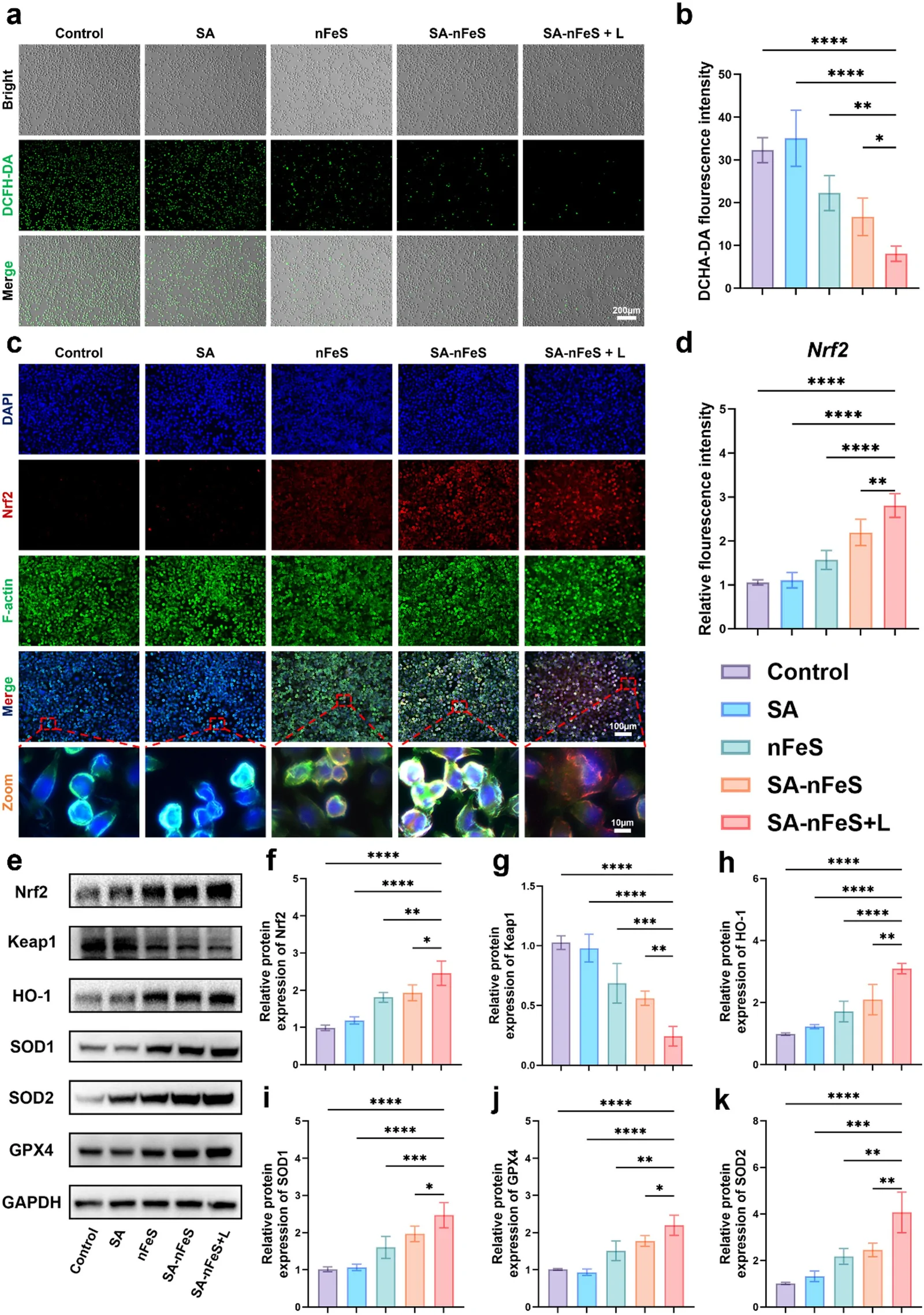
Intracellular ROS scavenging and activation of the Keap1/Nrf2 antioxidant defense pathway by the hydrogels. (a) Representative fluorescence images showing intracellular ROS levels detected using the DCFH-DA probe after different treatments. Scale bar = 200 μm. (b) Quantitative analysis of the DCFH-DA relative fluorescence intensity. (c) Representative immunofluorescence images showing the expression and nuclear translocation of Nrf2 (blue: DAPI-stained nuclei, red: F-actin, green: Nrf2). The zoomed-in panels highlight the colocalization of Nrf2 and nuclei. Scale bars = 100 μm and 10 μm (Zoom). (d) Quantitative analysis of relative Nrf2 fluorescence intensity. (e) Western blot bands of Nrf2, Keap1, HO-1, SOD1, SOD2, GPX4, and GAPDH (internal control) across different treatment groups. (f–k) Quantitative densitometric analysis of the relative protein expression levels of (f) Nrf2, (g) Keap1, (h) HO-1, (i) SOD1, (j) GPX4, and (k) SOD2, normalized to GAPDH. (Data are presented as mean ± standard deviation. * p < 0.05, ** p < 0.01, *** p < 0.001, **** p < 0.0001). (For interpretation of the references to colour in this figure legend, the reader is referred to the web version of this article.)

Persistent oxidative stress is a primary driver of chronic inflammation in the diabetic wound microenvironment, severely hindering tissue regeneration. To evaluate the intracellular ROS-scavenging capability of the hydrogels, we utilized a DCFH-DA fluorescent probe on cells subjected to oxidative stress. As shown in Fig. 4a and quantified in Fig. 4b, cells in the control and pure SA groups exhibited intense green fluorescence, indicating high levels of intracellular ROS accumulation. In contrast, treatment with nFeS-containing formulations significantly attenuated the green fluorescence. Notably, the SA-nFeS + L group demonstrated the most efficient ROS clearance (**** *p* < 0.0001 compared to the Control). This suggests that near-infrared (NIR) laser irradiation accelerates the release of active components from nFeS, maximizing its antioxidant efficacy.

To elucidate the underlying molecular mechanisms of this potent antioxidant effect, we investigated the Keap1/Nrf2 signaling pathway, a master regulator of cellular defense against oxidative stress. Immunofluorescence staining revealed that the SA-nFeS + L treatment dramatically upregulated Nrf2 expression (Fig. 4c). More importantly, as evident in the magnified panels (Zoom), the Nrf2 signal (green) strongly colocalized with the DAPI-stained nuclei (blue), confirming the successful nuclear translocation of Nrf2. Quantitative analysis further verified that the SA-nFeS + L group achieved the highest relative Nrf2 fluorescence intensity among all groups (Fig. 4d).

We subsequently conducted western blot analysis to evaluate the expression levels of key proteins within this pathway (Fig. 4e). Compared to the Control group, the SA-nFeS + L group exhibited a significant downregulation of the Nrf2-inhibitor Keap1 (Fig. 4g) and a concurrent robust upregulation of Nrf2 (Fig. 4f). Consequently, the downstream endogenous antioxidant enzymes, including heme oxygenase-1 (HO-1), superoxide dismutase 1 (SOD1), superoxide dismutase 2 (SOD2), and glutathione peroxidase 4 (GPX4), were all significantly upregulated (Fig. 4h–k). Collectively, these results convincingly demonstrate that the SA-nFeS hydrogel, particularly when triggered by mild photothermal stimulation, effectively mitigates oxidative stress by activating the endogenous Keap1/Nrf2 defense system. This vital antioxidant action paves the way for reprogramming the hostile inflammatory microenvironment into a pro-healing state.

### 3.5 Modulation of macrophage polarization and immune microenvironment reprogramming

**Fig. 5:**
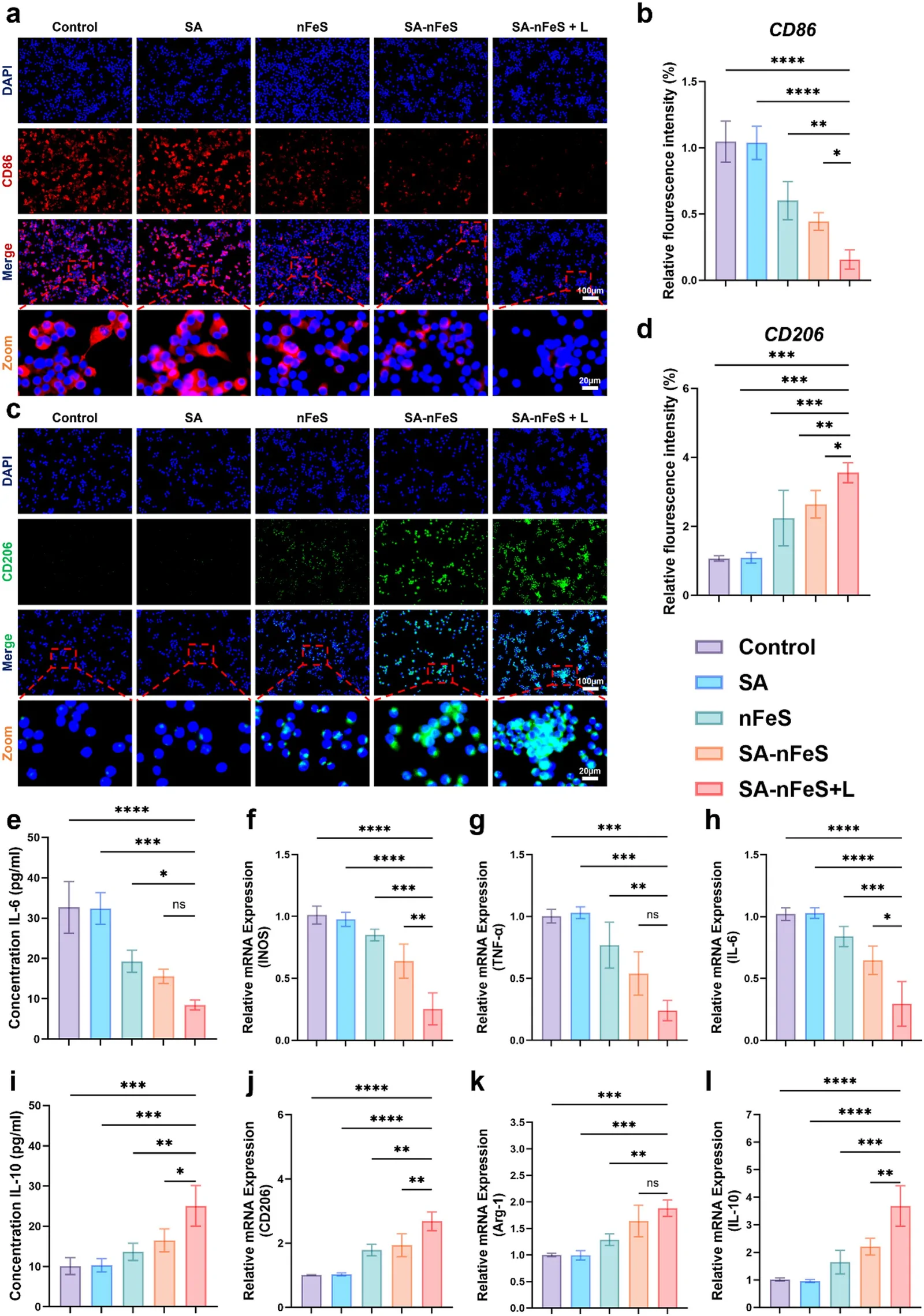
Reprogramming of macrophage polarization and immune microenvironment modulation by the hydrogels. (a) Representative immunofluorescence images of the M1 macrophage marker CD86 (red) and DAPI-stained nuclei (blue). The zoomed-in panels highlight cellular morphology. Scale bars = 100 μm and 20 μm (Zoom). (b) Quantitative analysis of the relative fluorescence intensity of CD86. (c) Representative immunofluorescence images of the M2 macrophage marker CD206 (green) and DAPI-stained nuclei (blue). Scale bars = 100 μm and 20 μm (Zoom). (d) Quantitative analysis of the relative fluorescence intensity of the M2 marker (CD206). (e, i) ELISA quantification of the secretion levels of (e) pro-inflammatory IL-6 and (i) anti-inflammatory IL-10 in the culture supernatant. (f–h) Relative mRNA expression levels of pro-inflammatory markers: (f) iNOS, (g) TNF-α, and (h) IL-6, determined by RT-qPCR. (j–l) Relative mRNA expression levels of anti-inflammatory markers: (j) CD206, (k) Arg-1, and (l) IL-10, determined by RT-qPCR. (Data are presented as mean ± standard deviation. ns: no significant difference; * p < 0.05, ** p < 0.01, *** p < 0.001, **** p < 0.0001). (For interpretation of the references to colour in this figure legend, the reader is referred to the web version of this article.)

The transition of macrophages from a pro-inflammatory (M1) phenotype to an anti-inflammatory (M2) phenotype is a critical checkpoint for resolving chronic inflammation and initiating tissue regeneration in diabetic wounds. Building upon the robust ROS-scavenging and Nrf2-activating capabilities of the SA-nFeS hydrogel, we further investigated its immunomodulatory effects on macrophages. Immunofluorescence staining was performed to evaluate the phenotypic switch. As depicted in Fig. 5a and its quantification (Fig. 5b), cells in the Control and pure SA groups exhibited high expression of CD86 (a typical M1 marker, red fluorescence). Conversely, the SA-nFeS + L treatment drastically suppressed CD86 expression (**** *p* < 0.0001). Concurrently, the expression of the M2 macrophage marker CD206 (green fluorescence) was profoundly upregulated in the SA-nFeS + L group (Fig. 5c, d), indicating a successful phenotypic shift driven by the synergistic photothermal and iron-release mechanisms.

To functionally validate this immune microenvironment reprogramming, we conducted extensive cytokine profiling using ELISA and quantitative real-time PCR (RT-qPCR). The SA-nFeS + L hydrogel significantly downregulated the secretion (Fig. 5e) and mRNA transcription (Fig. 5h) of the pro-inflammatory cytokine IL-6. Similarly, the mRNA expression levels of other key M1 markers, including iNOS (Fig. 5f) and TNF-α (Fig. 5g), were remarkably reduced. In stark contrast, the production of the anti-inflammatory cytokine IL-10 was significantly elevated at both the protein (Fig. 5i) and mRNA (Fig. 5l) levels. Furthermore, the mRNA expression of essential M2 polarization markers, CD206 (Fig. 5j) and Arg-1 (Fig. 5k), peaked in the SA-nFeS + L group. Collectively, these results provide compelling evidence that the sSA-nFeS hydrogel effectively mitigates the hostile inflammatory microenvironment by reprogramming macrophages toward a pro-healing M2 phenotype, establishing a highly favorable immunological foundation for diabetic wound repair.

### 3.6 *In vivo* assessment of diabetic wound healing

**Fig. 6:**
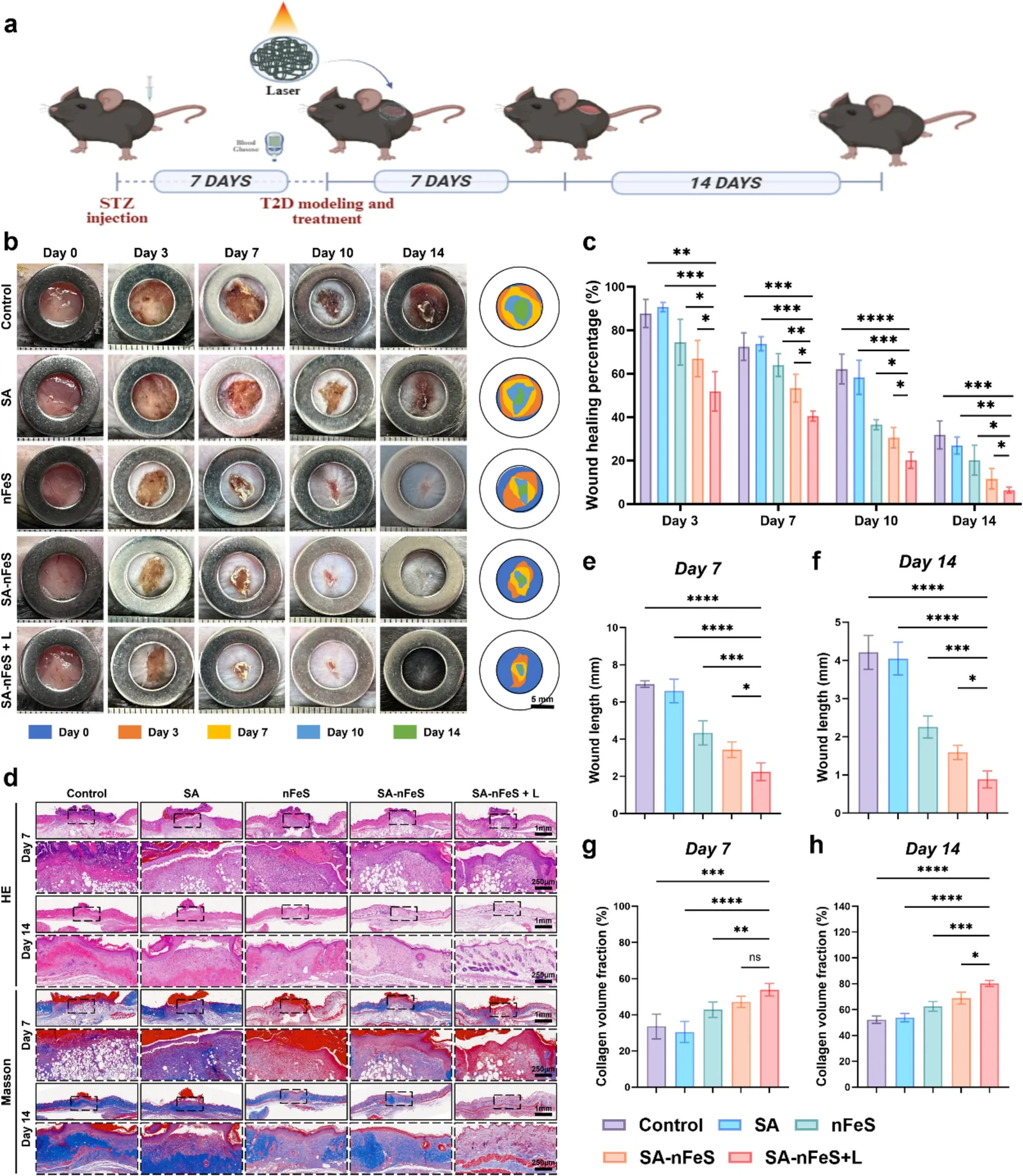
*In vivo* evaluation of the SA-nFeS hydrogel in promoting diabetic wound healing. (a) Schematic illustration of the STZ-induced diabetic mouse model and the *in vivo* wound treatment timeline. (b) Representative macroscopic photographs of the diabetic wounds in different treatment groups at Days 0, 3, 7, 10, and 14, accompanied by schematic tracings of the wound beds. Scale bar = 5 mm. (c) Quantitative analysis of the wound healing percentage over time. (d) Representative H&E and Masson's trichrome staining images of the wound tissues harvested at Day 7 and Day 14. The black dashed boxes indicate the wound gap or specific regions of tissue regeneration. Scale bars = 1 mm (low magnification) and 250 μm (high magnification). (e, f) Quantitative measurements of the wound length at (e) Day 7 and (f) Day 14 based on the H&E histological sections. (g, h) Quantitative analysis of the collagen volume fraction based on Masson's trichrome staining at (g) Day 7 and (h) Day 14. (Data are presented as mean ± standard deviation. ns: no significant difference; * p < 0.05, ** p < 0.01, *** p < 0.001, **** p < 0.0001).

To evaluate the *in vivo* therapeutic efficacy of the smart hydrogel, a full-thickness skin defect model was established in streptozotocin (STZ)-induced diabetic mice (Fig. 6a). The macroscopic progression of wound closure was monitored over a 14-day period. As shown in the representative photographs and wound area traces (Fig. 6b), the SA-nFeS + L group exhibited the most rapid contraction and re-epithelialization throughout the treatment process. Quantitative analysis of the remaining wound area revealed that by Day 14, the SA-nFeS + L group achieved nearly complete closure, with a reduction in wound area of over 90%, significantly outperforming the pure SA, non-irradiated SA-nFeS, and Control groups (****p < 0.0001) (Fig. 6c). This accelerated closure was further corroborated by the wound length measurements at Day 7 and Day 14 (Fig. 6e, f), demonstrating the potent synergistic effect of photothermal stimulation and nFeS-mediated microenvironment regulation.

To further investigate the quality of tissue regeneration, histological analyses were performed using Hematoxylin and Eosin (H&E) and Masson's trichrome staining on wound tissues harvested at Day 7 and Day 14 (Fig. 6d). At Day 7, H&E staining revealed severe inflammatory infiltration and wide wound beds in the Control group. In stark contrast, the SA-nFeS + L group showed markedly reduced inflammation and thicker, more organized granulation tissue. By Day 14, the SA-nFeS + L treated wounds exhibited complete continuous epidermal layers and evident regeneration of skin appendages, such as hair follicles, closely resembling healthy native skin.

Furthermore, collagen deposition—a crucial hallmark of the tissue remodeling phase—was evaluated using Masson's trichrome staining, where collagen fibers are stained blue. The SA-nFeS + L group demonstrated the most robust and densely packed collagen networks at both Day 7 and Day 14. Quantitative analysis of the collagen volume fraction confirmed that the SA-nFeS + L group reached the highest levels of collagen deposition (approximately 80% at Day 14) (Fig. 6g, h). These histological findings strongly indicate that the SA-nFeS + L hydrogel effectively transitions the stalled diabetic wound out of the chronic inflammatory phase and orchestrates a highly efficient proliferative and remodeling response.

### 3.7 *In vivo* verification of immune microenvironment reprogramming and antioxidant defense activation

The pathological microenvironment of diabetic wounds is characterized by persistent oxidative stress and chronic inflammation, which stall the healing process. To verify that the accelerated wound closure observed *in vivo* is driven by the synergistic modulation of this microenvironment, we evaluated the *in vivo* macrophage polarization and the activation state of the endogenous antioxidant defense system using immunofluorescence staining of the wound tissues at Day 7 and Day 14.

**Fig. 7:**
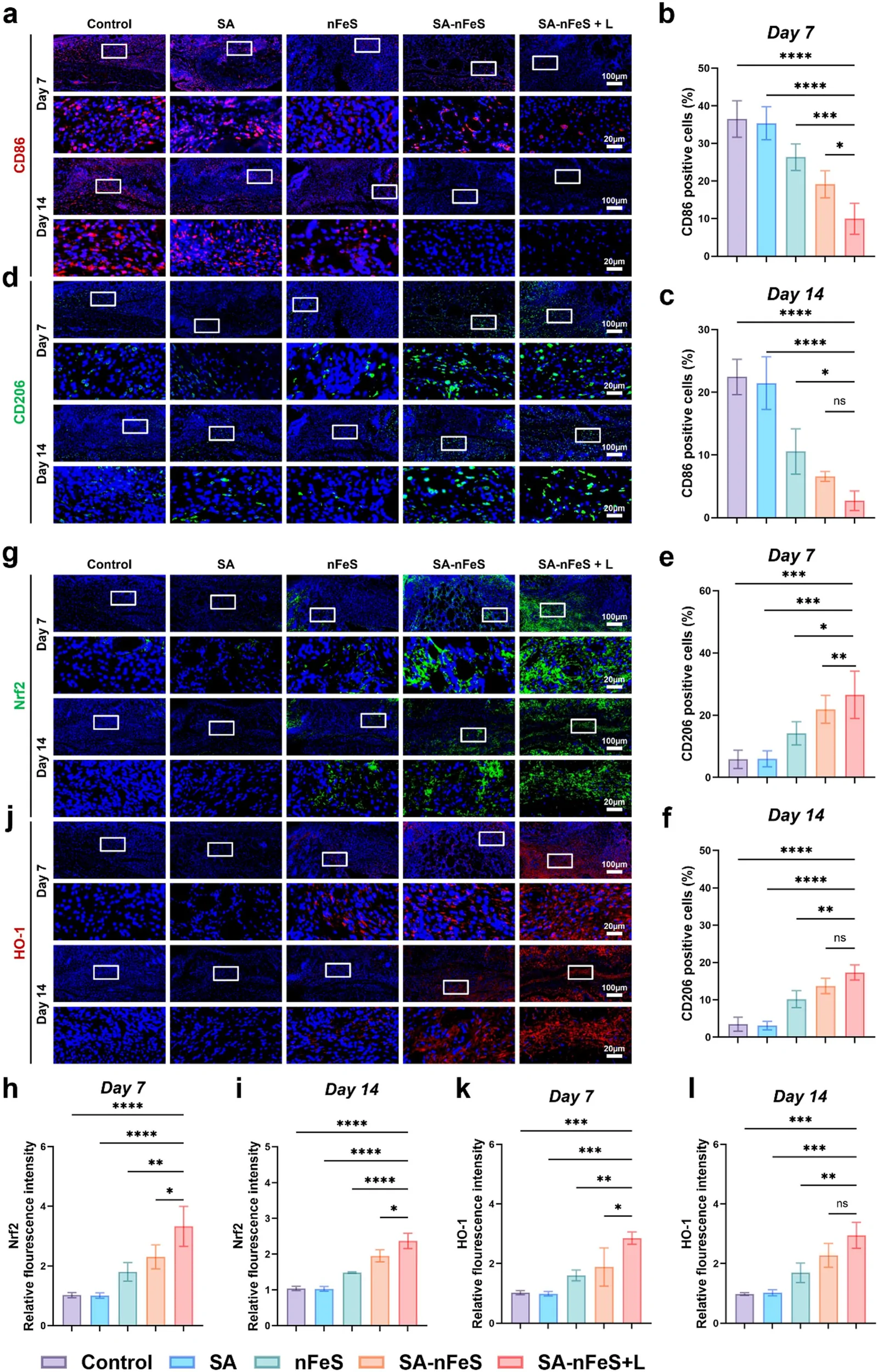
*In vivo* verification of immune microenvironment reprogramming and activation of the Nrf2/HO-1 antioxidant pathway. (a) Representative immunofluorescence images of the M1 macrophage marker CD86 (red) and DAPI-stained nuclei (blue) in wound tissues at Day 7 and Day 14. The white boxes indicate the magnified regions shown below. Scale bars = 100 μm and 20 μm (high magnification). (b, c) Quantitative analysis of the percentage of CD86-positive cells at (b) Day 7 and (c) Day 14. (d) Representative immunofluorescence images of the M2 macrophage marker CD206 (green) and DAPI (blue). Scale bars = 100 μm and 20 μm. (e, f) Quantitative analysis of the percentage of CD206-positive cells at (e) Day 7 and (f) Day 14. (g) Representative immunofluorescence images of Nrf2 (green) and DAPI (blue). Scale bars = 100 μm and 20 μm. (h, i) Quantitative analysis of relative Nrf2 fluorescence intensity at (h) Day 7 and (i) Day 14. (j) Representative immunofluorescence images of HO-1 (red) and DAPI (blue). Scale bars = 100 μm and 20 μm. (*k*,*l*) Quantitative analysis of relative HO-1 fluorescence intensity at (k) Day 7 and (l) Day 14. (Data are presented as mean ± standard deviation. ns: no significant difference; * p < 0.05, ** p < 0.01, *** p < 0.001, **** p < 0.0001). (For interpretation of the references to colour in this figure legend, the reader is referred to the web version of this article.)

Macrophage plasticity is critical for transitioning the wound from the inflammatory phase to the proliferative phase. We assessed the expression of CD86 (an M1 pro-inflammatory macrophage marker) and CD206 (an M2 anti-inflammatory macrophage marker). As shown in Fig. 7a, tissues from the Control and pure SA groups exhibited extensive CD86-positive signal (red) at both Day 7 and Day 14. In contrast, the SA-nFeS + L hydrogel dramatically suppressed CD86 expression, as confirmed by quantitative analysis (Fig. 7b, c). Concurrently, the expression of the M2 marker CD206 (green) was robustly elevated in the SA-nFeS + L group (Fig. 7d–f). These findings confirm that the SA-nFeS + L system successfully reprograms the hostile wound microenvironment from a state of chronic inflammation to one conducive to regeneration *in vivo*.

Furthermore, to validate the *in vivo* activation of the antioxidant defense system, we evaluated the expression of Nrf2 and its downstream effector, heme oxygenase-1 (HO-1). As depicted in Fig. 7g and quantified in Fig. 7h, i, the Nrf2 fluorescence intensity (green) was significantly enhanced in the SA-nFeS + L group at both time points compared to the control groups. Consequently, the expression of the antioxidant enzyme HO-1 (red) was also remarkably upregulated (Fig. 7j–l). These *in vivo* results perfectly align with our *in vitro* findings, firmly demonstrating that the SA-nFeS hydrogel activates the Keap1/Nrf2 signaling pathway, upregulating endogenous antioxidant enzymes and thereby attenuating oxidative stress within the diabetic wound bed.

### 3.8 *In vivo* systemic biosafety evaluation

Systemic safety and biocompatibility are paramount prerequisites for the clinical translation of nanomaterial-integrated wound dressings. To comprehensively evaluate the potential off-target toxicity of the hydrogels, major organs including the heart, liver, spleen, lung, and kidney were harvested from the mice at the end of the 14-day treatment period for extensive histological examination. As shown in Fig. S4, Hematoxylin and Eosin (H&E) staining revealed no observable morphological abnormalities, cellular necrosis, or significant inflammatory cell infiltration in any of the treatment groups compared to the healthy Control group. Specifically, the cardiac muscle fibers remained intact, the hepatocytes were arranged neatly without signs of steatosis, the splenic white and red pulps maintained their normal architecture, the alveolar septa in the lungs were not thickened, and the renal glomeruli and tubules exhibited typical healthy morphologies. Furthermore, Masson's trichrome staining was performed to assess potential fibrotic damage. Across all groups, including the SA-nFeS + L group, there was no abnormal collagen accumulation or fibrotic lesions observed in these critical organs. Collectively, these histological findings conclusively demonstrate that the SA-nFeS hydrogel, alongside the near-infrared laser irradiation and the subsequent localized release of iron ions, does not elicit any systemic toxicity, verifying its exceptional *in vivo* biosafety for practical clinical applications.

### 3.9 Clinical relevance and limitations

From a clinical perspective, wound dressings are selected according to wound depth, exudate level, infection status, and the requirement to maintain an appropriate moist healing environment. Conventional gauze, foam, hydrocolloid, alginate, and standard hydrogel dressings primarily provide physical protection, moisture balance, and exudate management, and current clinical evidence has not established consistent superiority of any single dressing class for diabetic wound healing (Eriksson et al., 2022). Negative-pressure wound therapy can support exudate removal and granulation-tissue formation in selected postoperative diabetic foot wounds, but its application depends on dedicated equipment, trained care, and available healthcare resources (Borys et al., 2019).

In comparison, the SA-nFeS hydrogel developed in this study is designed to combine a hydrated alginate matrix with hydrogen peroxide-responsive degradation, ROS attenuation, Nrf2-associated endogenous antioxidant activation, and modulation of macrophage-associated inflammatory phenotypes. The NIR-responsive release of iron-containing components may provide an additional degree of external control over material activity. These properties represent potential functional advantages over dressings that mainly provide passive wound coverage and exudate management. However, they should not be interpreted as evidence of clinical superiority, because no direct comparison with commercially available wound dressings was performed in the present study.

Several limitations should also be acknowledged. First, the therapeutic strategy requires NIR irradiation, and the penetration depth, optimal irradiation parameters, and feasibility of repeated treatment in human wounds remain to be established. The wound-surface temperature was not directly monitored during *in vivo* irradiation, and therefore the local thermal safety profile requires further investigation. In addition, the local temperature of the hydrogel and wound surface was not directly monitored during *in vivo* NIR irradiation. Therefore, the maximum *in situ* temperature and its relationship to clinically acceptable thermal ranges could not be determined. Future studies should incorporate real-time thermal monitoring and systematically optimize the irradiation power, duration, and treatment frequency to balance therapeutic responsiveness with tissue safety. Second, the present study used a non-infected diabetic full-thickness wound model without exogenous bacterial inoculation. Accordingly, *in vivo* bacterial burden and antibacterial efficacy were not evaluated. Future studies using clinically relevant infected wound models, including multidrug-resistant bacterial infections, are required to determine whether the SA-nFeS hydrogel provides therapeutic benefits under infectious conditions. Third, the observation period was limited to 14 days, and systemic safety was assessed primarily by histological examination of major organs. Long-term studies are therefore required to characterize hydrogel degradation, iron metabolism, potential cumulative toxicity, and tissue responses. Finally, direct comparisons with commercial dressings, validation in large-animal models, and well-designed clinical studies will be necessary before the potential clinical benefits of this system can be established.

## CRediT authorship contribution statement

**Xiaofang Wang:** Writing – original draft, Formal analysis, Data curation. **Jun Chen:** Writing – original draft, Data curation. **Tingyi Guo:** Writing – original draft, Formal analysis, Data curation. **Huisheng Yao:** Data curation. **Nan Li:** Investigation. **Zhenggang Li:** Writing – review & editing, Investigation. **Jia Li:** Writing – review & editing, Supervision, Project administration.

## Clinical trial number

Not applicable.

## Ethics approval and consent to participate

All animal experiments were performed in accordance with the ARRIVE guidelines and the institutional guidelines for the care and use of laboratory animals.

## Funding

This research did not receive any specific grant from funding agencies in the public, commercial, or not-for-profit sectors.

## Declaration of competing interest

The authors declare that they have no known competing financial interests or personal relationships that could have appeared to influence the work reported in this paper.

## Data Availability

All data and materials were included in this study and supplementary data

## References

[bb0005] Alven S., Peter S., Mbese Z., Aderibigbe B.A. (2022). Polymer-based wound dressing materials loaded with bioactive agents: potential materials for the treatment of diabetic wounds. Polymers.

[bb0010] Bei Z.W., Ye L., Tong Q., Ming Y., Yang T.Y., Zhu Y.Z., Zhang L.H., Li X.C., Deng H.Z., Liu J. (2024). Thermostimulated shrinking and adhesive hydrogel dressing for treating chronic diabetic wounds. Cell Rep. Phys. Sci..

[bb0015] Berthiaume F., Yarmush M., Hsia H., Nanda V., Dash B., D’Elia A., Kumar S., Kang H. (2021). Self-assembled elastin-like polypeptide fusion protein coacervates as competitive inhibitors of advanced glycation end-products enhance diabetic wound healing. J. Control. Release.

[bb0020] Borys S., Hohendorff J., Frankfurter C., Kiec-Wilk B., Malecki M.T. (2019). Negative pressure wound therapy use in diabetic foot syndrome—from mechanisms of action to clinical practice. Eur. J. Clin. Investig..

[bb0025] Cui J.D., Shi J.B., Liu Y.J., Shi X.B., Sun J., He Z.G., Luo C., Zhang S.W. (2025). Engineered hydrogel platform for diabetic wound healing. Chem. Eng. J..

[bb0030] Eriksson E., Liu P.Y., Schultz G.S., Martins-Green M.M., Tanaka R., Weir D., Gould L.J., Armstrong D.G., Gibbons G.W., Wolcott R. (2022). Chronic wounds: treatment consensus. Wound Repair Regen..

[bb0035] Fang M. (2018). Trends in the prevalence of diabetes among U.S. adults: 1999–2016. Am. J. Prev. Med..

[bib160] Feng S, Xuan Y, Jin H (2026). Nanozyme for precision treatment of hepatocellular carcinoma. Mater. Today Bio..

[bb0040] González P., Lozano P., Ros G., Solano F. (2023). Hyperglycemia and oxidative stress: an integral, updated and critical overview of their metabolic interconnections. Int. J. Mol. Sci..

[bb0045] Jin W.X., Li Y., Yu M.R., Ren D.Y., Han C.M., Guo S.X. (2025). Advances of exosomes in diabetic wound healing. Burns Trauma.

[bb0050] Li Q., Xia S.Z., Yin Y.T., Guo Y.P., Chen F.F., Jin P.S. (2018). miR-5591-5p regulates the effect of ADSCs in repairing diabetic wound via targeting AGEs/AGER/JNK signaling axis. Cell Death Dis..

[bb0055] Li X., Zhang W.J., Li H.L., Shuai Q., Zhang X.C., Pich A. (2025). Sprayed aqueous microdroplets for spontaneous synthesis of functional microgels. Angew. Chem. Int. Ed..

[bib161] Li X, Li Z, Liu J (2026). Engineering stem cell-based nanotherapeutics to overcome myocardial ischemia-reperfusion injury. Biomaterials.

[bb0060] Li X., Ouyang Z.J., Hetjens L., Ni M., Lin K.L., Hu Y., Shi X.Y., Pich A. (2025). Functional dendrimer nanogels for DNA delivery and gene therapy of tumors. Angew. Chem..

[bb0065] Li H.L., Song F.Y., Hu X.Y., Jing L., Shuai Q., Su W.K., Pich A., Li X. (2026). Tailored nanogel network topology enables clinical ultrasound-induced mechanochemical activation for in vivo therapy. Angew. Chem. Int. Ed..

[bib174] Liu C., Huang X., Chen K.S., Xiong S., Yaremenko A.V., Zhen X., Tao W. (2025). Systemic reprogramming of tumour immunity via IL-10-mRNA nanoparticles. Nature Nanotechnol..

[bb0070] Liu J., Qu M.Y., Wang C.R., Xue Y.M., Huang H., Chen Q.M., Sun W.J., Zhou X.W., Xu G.H., Jiang X. (2022). A dual-cross-linked hydrogel patch for promoting diabetic wound healing. Small.

[bib169] Liao J, Fan L, Li Y (2023). Recent advances in biomimetic nanodelivery systems: New brain-targeting strategies. J. Control Release.

[bib171] Liao J., He W., Li L., Wang J., Gong L., Zhang Q., Lin Z. (2025). Mitochondria in brain diseases: bridging structural-mechanistic insights into precision-targeted therapies. Cell Biomater..

[bib170] Liao J., He W., Zhang S., Wang J., Cheng H., Gong L., Lin Z. (2025). Magnetic field driven ceria nanosystems for mitochondria targeted therapy of ischemic stroke. Adv. Funct. Mater..

[bib162] Liao J, Huang Q, Li R, Gong L, Li T, Lin Z (2026). Long-acting nanomedicine for brain diseases. J Control Release.

[bib168] Liao J., Li Y., Fan L.I., Sun Y., Gu Z., Xu Q.Q., Lu Y. (2024). Bioactive ceria nanoenzymes target mitochondria in reperfusion injury to treat ischemic stroke. ACS Nano.

[bib159] Liao Jun, Lin Zhiqiang, Chuang Liu (2026). Precision uterine mRNA therapy to Restore implantation and fertility. Mater. Today.

[bib157] Liao J., Lin Z., Liu C. (2026). Programming extracellular protein fate with peptide chimeras. Chem.

[bib156] Liao J, Liu H, Li N (2026). Ligand-Triggered Topology Switching Converts Transient Recognition into Durable Nanofibrillar Anchoring. J. Am. Chem. Soc..

[bb0075] Liu S.P., Ge Z.H., Liu Y.P., Chen R., Xu J.X., Zhou Y.X., Sun M., Fan Z., Du J.Z. (2025). Iron homeostasis regulating glycopeptide hydrogel reprograms the healing process of diabetic wounds infected with MRSA. Adv. Funct. Mater..

[bib173] Liu C., Yaremenko A.V., Li X., You X., Zhu X., Liu N., Tao W. (2026). mRNA technology for the prevention and treatment of HIV-1 infection. Nature Rev. Bioeng..

[bb0080] Luo X., Yuan W.W., Xiang J.F., Tian Z.Q., Li G.X., Zheng C.G., Zhu D.M., Suo M., Lu Y., Li X. (2026). Global cold local photothermal-mediated synergistic metabolic antitumor immunity. Adv. Funct. Mater..

[bb0085] Mesfin J.M., Carrow K.P., Chen A., Hopps M.P., Holm J.J., Lyons Q.P., Nguyen M.B., Hunter J.D., Magassa A., Wong E.G. (2025). Protein-like polymers targeting Keap1/Nrf2 as therapeutics for myocardial infarction. Adv. Mater..

[bb0090] Qu M.Y., Xu W.Z., Zhou X.W., Tang F., Chen Q., Zhang X., Bai X.F., Li Z.Y., Jiang X., Chen Q.M. (2024). An ROS-scavenging treg-recruiting hydrogel patch for diabetic wound healing. Adv. Funct. Mater..

[bb0095] Shi S.Y., Hu L.M., Hu D., Ou X.H., Huang Y.S. (2024). Emerging nanotherapeutic approaches for diabetic wound healing. Int. J. Nanomedicine.

[bib166] Shi X, Zhao Y, Gao HY (2025). NFS1, together with FXN, protects cells from ferroptosis and DNA damage in diffuse large B-cell lymphoma. Redox Biol..

[bb0100] Simon M., Aristidis V., David J.M. (2021). Advanced bandages for diabetic wound healing. Sci. Transl. Med..

[bb0105] Sun W.J., Zhang X., Zhao Y.L., Ye B.X., Gao W., Jiang G.Y., Li X., Lin X.J. (2025). pH/NIR dual-responsive implantable patch for combined photothermal and chemotherapy of tumors. Int. J. Pharmaceut. X..

[bb0110] Tong S., Li Q.Y., Liu Q.Y., Song B., Wu J.Z. (2022). Recent advances of the nanocomposite hydrogel as a local drug delivery for diabetic ulcers. Front. Bioeng. Biotechnol..

[bib163] Wang H, Yu H, Bai X (2025). Recent advances in hydrogel-based delivery systems for the treatment of pancreatic cancer. Mater. Today Bio..

[bb0115] Wang H.Z., Zhang L.M. (2024). Intelligent biobased hydrogels for diabetic wound healing: a review. Chem. Eng. J..

[bb0120] Wang H.P., Wang X.N., Mao M.X., Chen X., Han Z.W., Xun Z.Y., Wang Q., Qi Y.L., Zhao W.T., Li T.Q. (2025). Oral iron sulfide prevents acute alcohol intoxication by initiating the endogenous multienzymatic antioxidant defense system. Sci. Adv..

[bb0125] Wei X.L., Liu C.K., Li Z.Q., Gu Z.X., Yang J.X., Luo K. (2024). Chitosan-based hydrogel dressings for diabetic wound healing via promoting M2 macrophage-polarization. Carbohydr. Polym..

[bib165] Xie C., Liao J., Li Y., Zhang Y., Xiao Z., Wang Y., Wang T. (2025). Synergistic anti-inflammatory effect of cascade nanozymes for neural recovery in ischemic stroke. Chinese Chem. Lett..

[bb0130] Xin P.K., Han S.Y., Huang J., Zhou C.L., Zhang J.Y., You X.R., Wu J. (2023). Natural okra-based hydrogel for chronic diabetic wound healing. Chin. Chem. Lett..

[bib172] Xu P., Liu M., Liu M., Shen A. (2025). Artificial intelligence in surgical oncology: a comprehensive review from preoperative planning to postoperative care. Intell. Oncol..

[bib164] Yang J., Guo L., Liao J., Yi H. (2024). Nano-enhanced nature medicine for ischemic stroke: opportunities and challenges. Biomed. Technol..

[bb0135] Yang Y.Y., Zhong S.L., Meng F.Y., Cui X.J. (2024). Multi-functional hydrogels to promote diabetic wound healing: a review. Chem. Eng. J..

[bib167] Zang J, Ren Z, Wang H (2025). Emerging Nanozyme Strategies for Precision Breast Cancer Treatment. Adv. Sci. (Weinh).

[bb0140] Zeng Q., Du S., Yuan R., Zeng Y., Li X.F., Wu Y.W., Chen K., Tao L., Tang Z.H., Deng X.L. (2025). Self-healing hydrogel dressing with solubilized flavonoids for whole layer regeneration of diabetic wound. Adv. Healthc. Mater..

[bb0145] Zhang J.H., Hu J.F., Chen B.S., Zhao T.B., Gu Z.P. (2021). Superabsorbent poly(acrylic acid) and antioxidant poly(ester amide) hybrid hydrogel for enhanced wound healing, Regenerative. Biomaterials.

[bb0150] Zhang X., Sun W.J., Li X., Li Y.Z., Xiang S., Zhang L.F., Yang Y.P. (2026). Cyano-modulated Zn (II)-Schiff base complexes for lysosome-targeted fluorescence imaging and pH-responsive camptothecin delivery. Int. J. Pharmaceut. X..

[bib158] Zhang S, Wang T, Gao T (2025). Imaging probes for the detection of brain microenvironment. Coll. Surf B Biointerf..

[bb0155] Zhao H., Huang J., Li Y., Lv X.J., Zhou H.T., Wang H.R., Xu Y.Y., Wang C., Wang J., Liu Z. (2020). ROS-scavenging hydrogel to promote healing of bacteria infected diabetic wounds. Biomaterials.

