# Supplementary material for: An NIR-responsive FeS-loaded alginate hydrogel promotes diabetic wound healing through Nrf2 activation and macrophage polarization
Source: Int J Pharm X. 2026 Aug 21;12:100645. doi: 10.1016/j.ijpx.2026.100645 (PMC13542600; doi:10.1016/j.ijpx.2026.100645)
Supplement: Supplementary file 1 [file mmc1.docx]

### **A Multifunctional FeS‑Loaded Hydrogel for Diabetic Wound Healing *via* Photothermal Triggered Bacterial Ferroptosis and Nrf2‑Activated Antioxidant Defense**

**Materials and Methods**

**1. Materials**

All chemicals were of analytical grade and used without further purification. Iron(III) chloride hexahydrate (FeCl₃·6H₂O), L‑cysteine, sodium alginate, calcium carbonate (CaCO₃), glucono‑δ‑lactone (GDL), 2,2‑diphenyl‑1‑picrylhydrazyl (DPPH), and 2,2′‑azinobis(3‑ethylbenzothiazoline‑6‑sulfonic acid) diammonium salt (ABTS) were purchased from Sigma‑Aldrich (USA). Potassium persulfate (K₂S₂O₈) was obtained from Aladdin (China). Phosphate‑buffered saline (PBS, 1×, pH 7.4) and Dulbecco’s modified Eagle’s medium (DMEM) were supplied by Gibco (USA). Hydrogen peroxide (H₂O₂, 30 wt%) was purchased from Sinopharm Chemical Reagent Co., Ltd. (China). Deionized water (DI water) with a resistivity of 18.2 MΩ·cm was produced using a Milli‑Q water purification system.

**Figure S1. The hydrodynamic diameter of the nFeS particles in aqueous dispersion (200 μg/mL) was measured by Dynamic Light Scattering (DLS).**

**Figure S2. ABTS scavenging efficiency at different FeS concentrations.**

**Figure S3. DPPH scavenging efficiency at different FeS concentrations.**

**Figure S4. *In vivo* systemic biosafety evaluation of the hydrogels.** Representative Hematoxylin and Eosin (H&E) and Masson's trichrome staining images of major organs (heart, liver, spleen, lung, and kidney) harvested from diabetic mice across different treatment groups after 14 days of wound healing. No noticeable pathological abnormalities, inflammation, or fibrotic lesions were observed. Scale bar = 200 μm.

**Table S1. Primer sequences used for Quantitative Real-Time PCR (RT-qPCR).**

| Gene Name | Forward Primer (5′ → 3′) | Reverse Primer (5′ → 3′) |
| --- | --- | --- |
| GAPDH | AGGTCGGTGTGAACGGATTTG | GGGGTCGTTGATGGCAACA |
| IL-6 | TAGTCCTTCCTACCCCAATTTCC | TTGGTCCTTAGCCACTCCTTC |
| TNF-α | CCCTCACACTCAGATCATCTTCT | GCTACGACGTGGGCTACAG |
| iNOS | GTTCTCAGCCCAACAATACAAGA | GTGGACGGGTCGATGTCAC |
| IL-10 | GCTCTTACTGACTGGCATGAG | CGCAGCTCTAGGAGCATGTG |
| CD206 | CTCTGTTCAGCTATTGGACGC | CGGAATTTCTGGGATTCAGCTTC |
| Arg-1 | CTCCAAGCCAAAGTCCTTAGAG | AGGAGCTGTCATTAGGGACATC |
